# Time-restricted eating improves health because of energy deficit and circadian rhythm: A systematic review and meta-analysis

**DOI:** 10.1016/j.isci.2024.109000

**Published:** 2024-01-26

**Authors:** Yuwen Chang, Tingting Du, Xiangling Zhuang, Guojie Ma

**Affiliations:** 1 Shaanxi Key Laboratory of Behavior and Cognitive Neuroscience, School of Psychology, Shaanxi Normal University, Xi’an, Shaanxi 710062, P.R. China

**Keywords:** Health sciences, Human metabolism, Nutrition

## Abstract

Time-restricted eating (TRE) is an effective way to lose weight and improve metabolic health in animals. Yet whether and how these benefits apply to humans is unclear. This systematic review and meta-analysis examined the effect of TRE in people with overweight and obesity statuses. The results showed that TRE led to modest weight loss, lower waist circumference and energy deficits. TRE also improved body mass index, fat mass, lean body mass, systolic blood pressure, fasting glucose levels, fasting insulin levels, and HbA1c%. Subgroup analysis demonstrated more health improvements in the TRE group than the control group under the *ad libitum* intake condition than in the energy-prescribed condition. Eating time-of-day advantages were only seen when there was considerable energy reduction in the TRE group than the control group (*ad libitum* condition), implying that the benefits of TRE were primarily due to energy deficit, followed by alignment with eating time of day.

## Highlights

• TRE leads to weight loss and metabolic improvements compared to the control group
• TRE improves health better with energy deficit (energy not prescribed)
• Energy deficit affects health improvements in TRE more than eating time of day
• E-TRE is better than d-TRE only with sufficient energy deficit

## Graphical abstract

**Figure:**
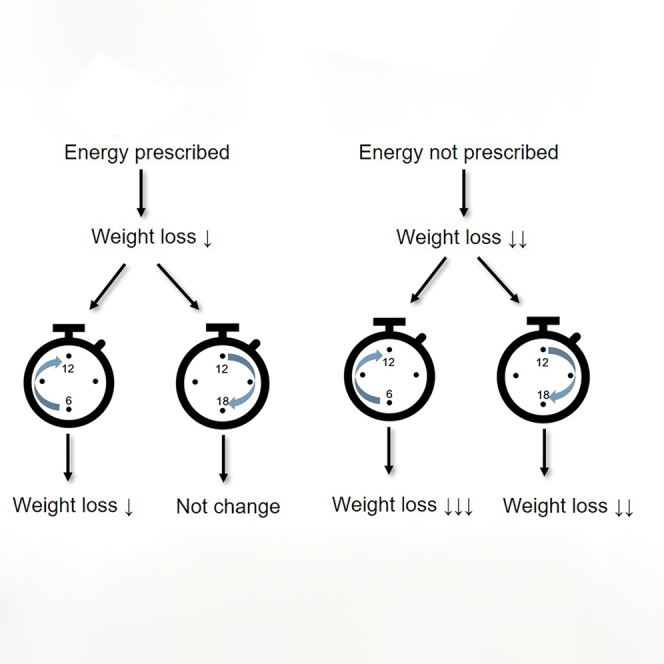

## Introduction

The past 20 years have shown dramatic increases in the prevalence of overweight and obesity, as well as related chronic diseases, such as cardiovascular disease, diabetes, and some cancers related to the conditions.^1^ Therefore, obesity has become a serious public concern that threatens the global population’s health. Daily caloric restriction (CR), with and without exercise, is the most common treatment used to fight obesity.^2^ Despite its short-term utility for weight loss, long-term CR is challenged by homeostatic physiological adaptations to weight loss,^3^ acute episodes of hunger,^4^ and relatively poor adherence.^5^ Time-restricted eating (TRE), the latest emerging time-based diet strategy, in which food is consumed within a consistent 8- to 12-h interval, has been acknowledged by researchers.^6^^,^^7^^,^^8^ TRE is considered an intermittent CR strategy compared to continuous CR since it is assumed to unintentionally reduce caloric intake by limiting the eating window during the day. As a subset of the CR strategy, TRE is superior to continuous CR and other types of intermittent fasting due to the alignment of the eating window with the time of day.^9^ The concomitant reduction in energy intake^10^ and considerable adherence^11^ may facilitate long-term weight loss and metabolic improvements.^12^^,^^13^ The earliest studies on time-restricted feeding (TRF) demonstrated benefits to metabolic health and lifespan in animals,^7^^,^^14^^,^^15^^,^^16^ but the extent to which these findings from animal models are translatable to humans has not been determined.^5^

A growing body of literature has examined the potential benefits of TRE on weight loss and metabolic health in humans, but the findings have been mixed. Although most studies confirmed that TRE could reduce body weight, the weight loss extent remains unclear. For example, 60 participants with overweight or obesity status underwent 10-h TRE for eight weeks and lost 10.7 kg, approximately 8.5% of their initial body weight.^17^^,^^18^ However, other studies presented a modest weight loss, ranging from less than 2 kg to about 5 kg.^19^^,^^20^^,^^21^^,^^22^ More controversy exists on the improvements in metabolism, which has stimulated a lot of studies and led to mixed results. Some studies explored whether TRE could enhance metabolic health,^23^^,^^24^ and others attempted to understand whether these benefits could exist in the absence of energy restriction or were modulated by the eating time of day.^25^^,^^26^

One important consideration that may help explain the previously mixed findings is that TRE includes several intervention subtypes, which are not equally effective.^27^^,^^28^ Previous studies classified TRE as under isocaloric conditions or *ad libitum* intake conditions according to whether the energy intake was restricted and as early time restricted eating (e-TRE) or delay-time restricted eating (d-TRE) depending on whether the daily eating window was within the early or late time of day.^10^^,^^29^ E-TRE is generally believed to improve metabolic homeostasis by sustaining daily rhythms in the feeding and fasting cycle, while a misalignment between eating and rhythm (d-TRE) may increase hunger and impair metabolic health.^7^^,^^30^^,^^31^ The variation in intervention methods regarding energy intake and eating rhythm contributed to the high heterogeneity of TRE studies.

Given the inconsistent clinical findings, meta-analysis can be used to explore the actual effect of TRE on weight loss and metabolic health as it provides a more precise estimate of the treatment effect and may explain heterogeneity between studies.^32^^,^^33^ To our knowledge, only four systematic reviews and meta-analyses focused on TRE have been conducted.^29^^,^^34^^,^^35^^,^^36^ However, the previous analyses had limitations in selecting TRE studies with poor quality or inconsistent inclusion criteria. For instance, two of them included both people with overweight status and physical activity,^35^^,^^36^ and two of them contained TRE strategies with quite short intervention durations of seven or fewer days.^29^^,^^35^ One meta-analysis considered religious fasting like Ramadan,^34^ and three meta-analyses included non-randomized controlled trials (n-RCTs).^34^^,^^35^^,^^36^ Furthermore, the number of TRE on clinical trials has greatly increased in the past two years alone,^37^ providing an opportunity for examining whether, and if so, how TRE could benefit weight loss and metabolic health in humans through a systematic review and meta-analysis.

Most clinical studies of TRE have focused on its beneficial outcomes, but there has been no general agreement on the underlying mechanism of the effects of TRE on weight loss and metabolic health. Previous studies demonstrated that participants on TRE with *ad libitum* intake commonly reduced their calories by 7%–22%.^37^ Thus, it is unclear whether energy restriction and time restriction collectively contribute to weight loss and metabolic health.^38^ Swiatkiewicz et al.^39^ proposed that reduced energy intake may account for some beneficial effects of TRE on body weight and metabolic outcomes, but one study reported improvements in insulin sensitivity and blood pressure without energy restriction or weight loss.^40^ Taken together, it is unclear whether energy restriction induced by TRE protocols or the alignment of the eating window with the time of day leads to healthy outcomes.^4^^,^^27^^,^^39^

The present study attempted to explore the mechanism of TRE for the first time through subgroup analysis in the meta-analysis by classifying TRE into isocaloric vs. *ad libitum* intake and e-TRE vs. d-TRE based on different intervention approaches. We assume that in the TRE strategies, energy restriction contributes more than the eating time of day to weight loss and metabolic health, supposing participants obtained more health improvements under the *ad libitum* intake condition with reduced energy intake compared to the isocaloric condition and e-TRE improves health status more than d-TRE only in the former condition, while the advantage of the eating time of day did not exist once energy intake was prescribed (isocaloric condition). Considering the limitations of previous meta-analyses, we conducted a systematic review and meta-analysis to examine the effect of TRE on weight loss and metabolic health in people with overweight and obesity statuses based on RCT trials and further investigated the factors that accounted for the TRE benefits.

## Results

**Figure 1:**
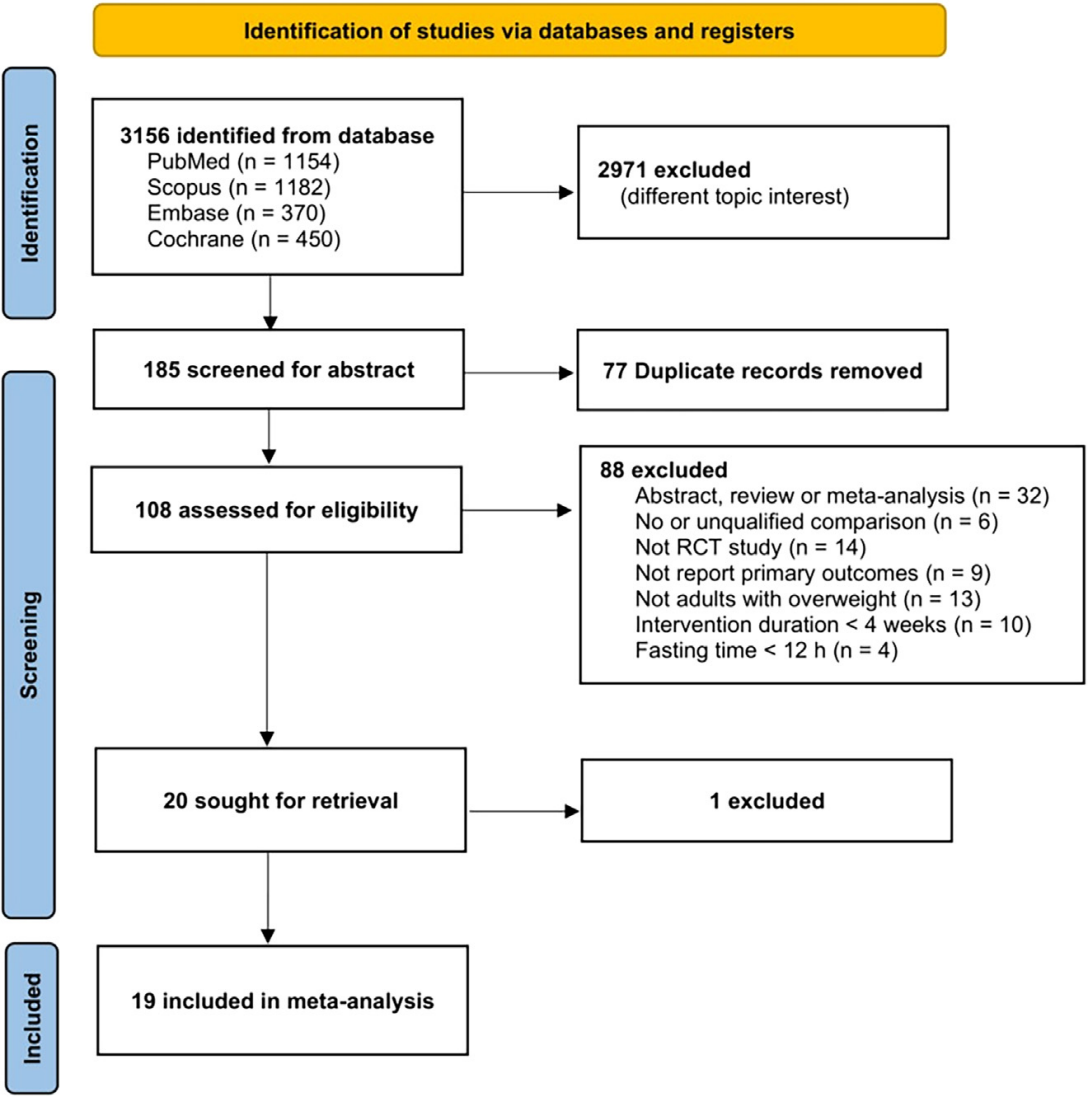
Scheme of study selection

Information related to the search strategy and number of participants is shown in Figure 1 and Table 1. The study characteristics are summarized in Table 2. The mean age of the participants ranged from 22.7 to 65 years, and the mean BMI ranged from 27.8 to 38.9 kg/m^2^. The eating window ranged from four to less than 12 h, and the experiment duration ranged from five to 48 weeks. Three of the studies^21^^,^^23^^,^^41^ were three-armed experiments containing two intervention groups and one control group, which we separately treated as two experiments in the data extraction and coding process.

**Table 1:** Participant information

	Intervention	Control	Total
Total number (N)	634	567	1201
Completed (n)	547	496	1043
Adherence (%)	86.28	87.48	86.84
Female gender (n/N, %)^a^	–	–	749/1151, 65.07

a One study did not report gender specifically,^20^ so we excluded this study when calculating the gender proportion. These studies only reported the total number of females in their samples. Thus, the number of females in the intervention and control groups was not available.

**Table 2:** Basic characteristics of the included studies

First author with year	Number (females/total)	Duration	Participant characteristics (year, kg/m^2^)	Energy intake	Intervention	Outcomes	Energy intake measurement
**Parallel-group studies**							

Gabel 2018	41/46	12 weeks	Age 49 ± 2.2 BMI 34.5 ± 1.1	ate *ad libitum*	8 h eating window from 10:00 to 18:00	WL, BMI, FM, BLM, VF; EI; SBP, DBP, TG, TC, LDL, HDL, FG, FI, HOMA-IR	A 7-day food record was collected at the weigh-in at baseline and week 12 and was reviewed by a nutrition professional
Chow 2020	17/20	12 weeks	Age 45.5 ± 12.1 BMI 34.1 ± 7.5	ate *ad libitum*	8 h self-selected eating window	WL, FM, BLM, VF; SBP, DBP, TG, HDL, LDL, FG, FI, HOMA-IR, HbA1c,	NI
Domaszewski 2020	45/45	6 weeks	Age 65 ± 5 BMI 28.1 ± 4.82	ate based on previous habits	8 h eating window from 12:00 to 20:00	WL, BMI, FM, BLM	NI
Cienfuegos 2020	34/38	8 weeks	Age 47.13 ± 2.8 BMI 36 ± 0.98	ate *ad libitum*	4 h eating window from 15:00 to 19:00	WL, FM, BLM; EI; SBP, DBP, TG, HDL, LDL, FG, FI, HbA1c	A 7-day food record was collected at the weigh-in at baseline and week 8 and was reviewed by a nutrition professional
Cienfuegos 2020^a^	36/39		Age 45.58 ± 2.63 BMI 36.58 ± 1.1		6 h eating window from 13:00 to 19:00		
Kunduraci 2020	36/70	12 weeks	Age 48.11 ± 2.23 BMI 34.67 ± 2.07	25% calorie reduction from habitual consumption	8 h self-selected eating window	WL, WC, BMI, FM, BLM; EI; SBP, DBP, TC, TG, HDL, LDL, FG, FI, HOMA-IR, HbA1c	A 24-h dietary recall method was used at baseline, weeks 4, 8, and 12, and analyzed with Nutrition Database Software System BeBIS
Lowe 2020	NA	12 weeks	Age 43.8 ± 11.2 BMI 31.4 ± 4	ate *ad libitum*	8 h eating window from 12:00 to 20:00	WL, WC, FM, BLM, VF; SBP, DBP, TG, TC, HDL, LDL, FI, FG, HbA1c, HOMA-IR, RMR	At-home weight measurements were used in a linear mathematical model of body weight dynamics to estimate energy intake
Peeke 2021	69/78	8 weeks	Age 44 ± 11 BMI 38.9 ± 7.7	500-1000 kcal/d deficit	8 h self-selected eating window	WL, FG	NI
Kotarsky 2021	18/21	8 weeks	Age 44 ± 7 BMI 29.6 ± 32.6	ate *ad libitum*	8 h eating window from 12:00 to 20:00	WL, WC, BMI, FM, BLM, VF; EI; SBP, DBP, HDL, FI, HbA1c	3-day dietary records at baseline and weeks 1, 4, and 7 were analyzed using Food Processor software
Ribeiro 2021	20/24	8 weeks	Age 32.2 ± 6.4 BMI 31.1 ± 4.6	20% calorie reduction from daily requirements	8 h eating window from 12:00 to 20:00	WL, WC, BMI, FM, BLM; TG, TC, LDL, HDL, FG, FI, HOMA-IR	NI
Che 2021	55/120	12 weeks	Age 48.5 ± 9.4 BMI >25	ate *ad libitum*	10 h eating window from 08:00 to 18:00	WL, BMI; EI; TG, TC, LDL, HDL, FG, FI, HOMA-IR, HbA1c	A 7-day dietary record at weigh-in at baseline and the endpoint were calculated by a nutritionist
Lin 2022	63/63	8 weeks	Age 52.2 ± 7.9 BMI 25.8 ± 3.7	1400 calories per day	8 h self-selected eating window	WL, WC, BMI, BLM, VF; EI; SBP, DBP, TG, TC, HDL, LDL, FG, FI, HOMA-IR	Participants recorded their daily food intake or took pictures and sent them to a dietitian
Liu 2022	68/139	12 months	Age 31.90 ± 9.02 BMI 31.55 ± 2.75	1500 to 1800 kcal/d for men, 1200 to 1500 kcal/d for women	8 h eating window from 8:00 to 16:00	WL, WC, BMI, FM, BLM; EI; SBP, DBP, TG, TC, HDL, LDL, FG, HOMA-IR	Participants’ daily dietary logs and food pictures were assessed using the Chinese Food Composition Table
Thomas 2022	68/81	12 weeks	Age 38 ± 7.8 BMI 34.1 ± 5.7	35% calorie reduction from REE	10 h eating window within 3 h of waking	WL, FM, BLM; EI	A 3-day dietary record at baseline and the endpoint was analyzed using Nutrition Data System for Research software
Queiroz 2022	22/30	8 weeks	Age 29.6 ± 6.14 BMI 30.45 ± 2.96	25% caloric intake below daily requirements	8 h eating window from 8:00 to 16:00	WL, BMI, FM, BLM; EI; TG, TC, LDL, HDL, FG, FI, HOMA-IR, RMR	Food photos of the three meals were sent daily to the nutritionist researchers
Queiroz 2022^a^	20/24		Age 27.83 ± 5.81 BMI 30.28 ± 2.94		8 h eating window from 12:00 to 20:00		
Ferrocino 2022	40/49	12 weeks	Age 56.9 ± 8 BMI 35.3 ± 3.1	500 -1000 kcal/d below REE	＜12 h self-selected eating window	WL, WC, BMI, FM, BLM; EI; SBP, DBP, TG, TC, HDL, FG, RMR	3-day food records at baseline and endpoint were analyzed using Win Food Pro 3 software
Jamshed 2022	72/90	14 weeks	Age 42.62 ± 10.78 BMI 39.62 ± 6.70	500 kcal below REE	8 h eating window from 7:00 to 15:00	WL, WC, FM, BLM; EI; SBP, DBP, TG, TC, HDL, LDL, FG, FI, HOMA-IR, HbA1c	A 3-day food record was kept using the Remote Food Photography Method
Zhang 2022	18/40	8 weeks	Age 22.99 ± 1 BMI 26.65 ± 1.59	ate *ad libitum*	6 h eating window from 7:00 to 13:00	WL, WC, BMI, FM, BLM, VF; EI; SBP, DBP, HR, TG, TC, HDL, LDL FG, FI, HOMA-IR, HbA1c	A 7-day food record at weight-in at baseline and the endpoint was analyzed using the China Food Composition Database
Zhang 2022^a^	18/39		Age 22.66 ± 0.71 BMI 28.16 ± 0.87		6 h eating window from 12:00 to 18:00		

**Crossover studies**							

Kahleova 2014	25/54	12 weeks	Age 59.4 ± 7 BMI 32.6 ± 4.9	500 kcal below REE	10 h eating window from 06:00 to 16:00	WL, WC, BMI; EI; SBP, DBP, TG, TC, HDL, LDL, FG, FI, RMR	Analysis of participants’ 3-day dietary records using the food-nutrient database NutriDan 1.2
Sutton 2018	0/8	5 weeks	Age 56 ± 9 BMI 32.2 ± 4.4	enough to maintain weight	6 h eating window with dinner before 15:00	WL	NI

WL, weight loss; WC, waist circumference; BMI, body mass index; FM, fat mass; BLM, lean body mass; VF, visceral fat; EI, energy intake; SBP, systolic blood pressure; DBP, diastolic blood pressure; HR, heart rate; TG, triglycerides; TC, total cholesterol; HDL, high-density cholesterol; LDL, low-density cholesterol; FG, fasting glucose; FI, fasting insulin; HOMA-IR, Homeostasis Model Assessment-Insulin Resistance; HbA1c, Hemoglobin A1c; RMR, resting metabolic rate; NI, no information; energy intake was not measured as an outcome; REE, energy resting expenditure.

Data on study characteristics, participants, dietary regimes, measurement of energy intake, and primary and secondary outcomes were extracted from the included studies. The macronutrient, consultation provided, physical exercise intensity, and methodology for measuring adherence were also extracted because they are vital factors for conducting a successful TRE intervention.^37^ For detailed information, see Table S3.

a This study contained two intervention groups and one control group, thus was coded and extracted as two separate trials with the same control group in this table.

A total of 3156 studies were screened. Of them, 2971 studies were excluded through abstract screening, and 77 duplicates were removed from the remaining 185 studies. Eighty-eight more studies were eliminated by reading the full texts. One study was excluded in the last retrieval because the participants were participating in active resistance training, although their average BMI was >25 kg/m^2^. Nineteen studies entered the final meta-analysis, with 11 studies in the isocaloric condition and the other eight in the *ad libitum* intake condition. Six studies involved e-TRE, seven studies involved d-TRE, and eight articles did not specify the eating time.

### Energy intake and eating window are associated with moderate weight loss

**Figure 2:**
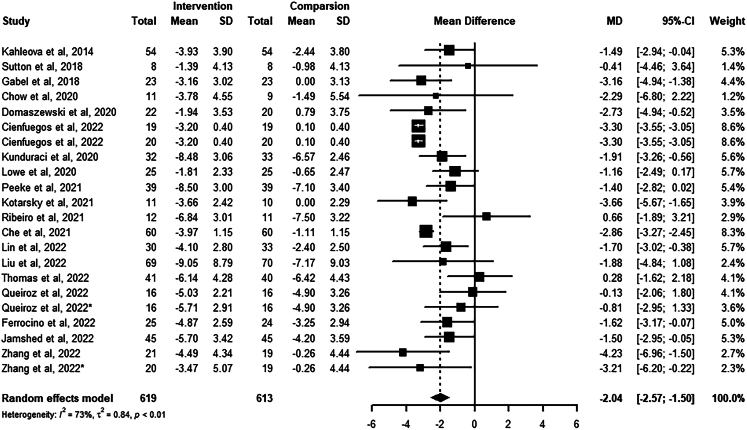
Meta-analysis results of weight loss

The percentage weight change^42^ from baseline to the endpoint between the intervention and control groups was analyzed in 19 studies with 22 arms (Figure 2). TRE effectively reduced body weight percentage compared to the control group (−2.04%, 95% confidence interval (CI): −2.57 to −1.50; low certainty evidence). Meta-regression was conducted to explore the relationship between energy intake, eating window, intervention duration, and weight loss percentage. Energy intake (*Z* = 5.23, p < 0.001) and eating window (*Z* = 2.26, p = 0.024) were significantly associated with weight loss percentage but not the intervention duration length (*Z* = 0.36, p = 0.72). No significant publication bias was found (Egger’s test p = 0.93).

The forest plot represents the effect of TRE on weight change percentage compared to the non-TRE group. The number of participants (Total), the mean difference (Mean), and the standard deviations (SD) of the primary outcomes from baseline to the endpoint for the intervention and control groups were used to calculate the mean difference and 95% confidence interval of weight change in the included studies. The random effects model was used to estimate the pooled effect size and account for possible heterogeneity.

### TRE, with or without energy prescription, improves waist circumference and body compositions

TRE, with or without caloric prescription, reduced waist circumference (−2.42 cm, 95%CI: −3.42 to −1.42, low certainty evidence), body mass index (−0.78 kg/m^2^, 95%CI: −1.01 to −0.54, moderate certainty evidence), and fat mass (−1.36 kg, 95%CI: −1.76 to −0.97, very low certainty evidence) but not visceral fat (−0.04 kg, 95%CI -0.10 to 0.01, very low certainty evidence) compared to the control group in other primary outcomes (Table 3 and Figure S1). However, TRE also slightly reduced lean body mass (−0.43 kg, 95%CI: −0.77 to −0.08, very low certainty evidence).

**Table 3:** Results of meta-analysis of outcomes

	*N*_*I*_*/N*_*C,*_*MD* with 95%CI	Certainty of evidence^a^
WL (%)	619/613, −2.04 (−2.57, −1.50)	low
WC (cm)	344/343, −2.42 (−3.42, −1.42)	low
BMI (kg/m^2^)	411/408, −0.78 (−1.01, −0.54)	moderate
FM (kg)	428/418, −1.36 (−1.76, −0.97)	very low
BLM (kg)	459/455, −0.43 (−0.77, −0.08)	very low
VF (kg)	145/145, −0.04 (−0.10, 0.01)	very low
EI (kcal)	502/500, −201.77 (−304.12, −99.43)	low
SBP (%)	351/348, −2.42 (−4.34, −0.50)	low
DBP (%)	351/348, −1.39 (−3.78, 1.01)	low
HR (bpm)	228/224, 0.47 (−1.06, 2.01)	very low
TG (mg/dL)	498/495, −4.37 (−9.32, 0.57)	low
TC (mg/dL)	448/448, 1.34 (−2.51, 5.19)	low
HDL (mg/dL)	509/505, 0.40 (−0.53, 1.34)	very low
LDL (mg/dL)	473/471, 1.96 (−0.95, 4.88)	very low
FG (mg/dL)	537/534, −2.57 (−4.73, −0.42)	very low
FI (μIU/mL)	415/411, −1.81 (−3.24, −0.38)	very low
HOMA-IR	264/258, −0.11 (−0.31, 0.09)	moderate
HbA1c (%)	380/379, −0.34 (−0.60, −0.07)	very low
RMR (kcal)	136/135, 19.54 (−1.61, 40.70)	very low

WL, weight loss; EI, energy intake; WC, waist circumference; BMI, body mass index; FM, fat mass; BLM, lean body mass; VF, visceral fat; SBP, systolic blood pressure; DBP, diastolic blood pressure; HR, heart rate; TG, triglycerides; TC, total cholesterol; HDL, high-density cholesterol; LDL, low-density cholesterol; FG, fasting glucose; FI, fasting insulin; HOMA-IR, Homeostasis Model Assessment-Insulin Resistance; HbA1c, Hemoglobin A1c; RMR, resting metabolic rate.

a The Grading of Recommendations Assessment; Development and Evaluation (GRADE) was assessed according to the Cochrane Handbook, the GRADE handbook and other articles.^43^ For detailed information, see Table S6. For risk of bias assessment see Tables S4 and S5.

### TRE reduces energy intake intentionally or unintentionally

**Figure 3:**
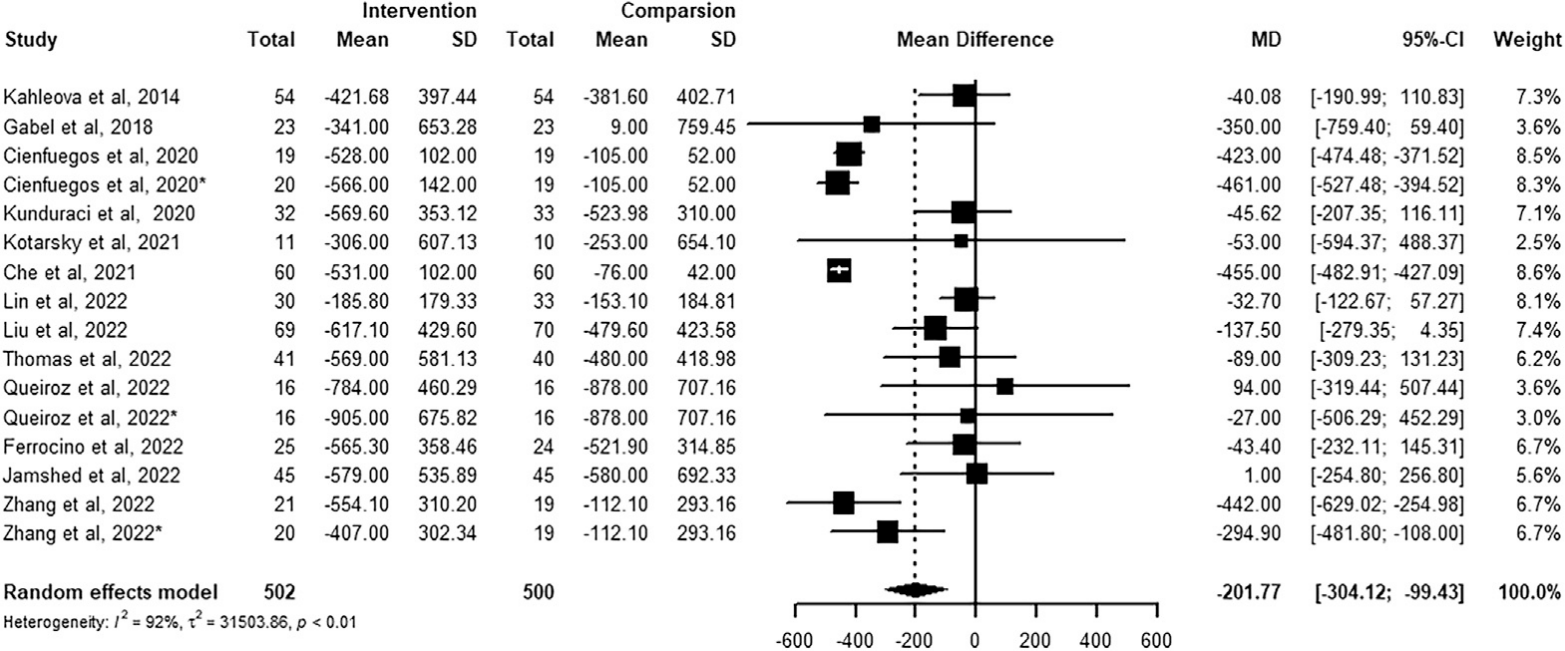
Meta-analysis results of energy intake

The change in energy intake from baseline to the endpoint between the intervention and control groups was analyzed in 13 studies with 16 arms (Figure 3). Individuals in the TRE group reduced energy intake by 201.77 kcal/day intentionally or unintentionally compared to the control group (−201.77 kcal, 95%CI: −304.12 to −99.43; low certainty evidence). Meta-regression found that the length of the eating window (*Z* = 2.05, p = 0.040) significantly affected energy intake but not the intervention duration (*Z* = 0.57, p = 0.57). There was no significant publication bias in the reporting of energy intake (Egger’s test p = 0.92).

The forest plot represents the effect of TRE on actual energy intake compared to the non-TRE group. The number of participants (Total), the mean difference (Mean), and the standard deviations (SD) of the primary outcomes from baseline to the endpoint for the intervention and control groups were used to calculate the mean difference and 95% confidence interval of the actual intake in the included studies. The random effects model was used to estimate the pooled effect size and account for possible heterogeneity.

### TRE, with or without energy prescription, improves systolic blood pressure and glycemic levels

In the metabolic risk factors (Table 3) analyzed, TRE, with or without energy prescription, effectively improved systolic blood pressure (−2.42%, 95%CI: −4.34 to −0.50, low certainty evidence), fasting glucose levels (−2.57 mg/dL, 95%CI: −4.73 to −0.42, very low certainty evidence), fasting insulin levels (−1.81 μIU/mL, 95%CI: −3.24 to −0.38, very low certainty evidence), and HbA1c levels (−0.34, 95%CI: −0.60 to −0.07, very low certainty evidence) but did not benefit other metabolic parameters compared to the control group.

### Energy reduction brings more benefits than isocaloric diets in TRE strategies

To test the crucial effect of actual energy consumption on the TRE diet, we classified the TRE regime as an isocaloric condition (energy prescribed) versus an *ad libitum* intake condition (energy not prescribed) to conduct a subgroup analysis (Table 4). In the analysis of weight loss percentage and energy intake, individuals lost 3.08% body weight (−3.08%, 95%CI: −3.42 to −2.73) compared to the control group under the *ad libitum* condition and showed a lower percentage of weight loss under the isocaloric condition (−1.25%, 95%CI: −1.74 to −0.75). Compared to the considerable energy reduction between the TRE and control groups under the *ad libitum* condition (−445.96 kcal, 95%CI: −468.58 to −423.33), there was no significant energy reduction in the TRE group in the isocaloric condition (−51.53 kcal, 95%CI: −106.62 to 3.55). Taken together, we attributed the weight loss under the isocaloric condition to the benefits of alignment with the eating time of day since the energy intake was not different in this condition. Yet the fact that considerable energy deficit (−445.96 kcal/d) and relatively modest body weight loss (−3.08%) in the *ad libitum* condition was confusing. We then used the Body Weight Planner (niddk.nih.gov/bwp) in NIH (National Institute of Health) to compare the anticipated and actual weight loss given the energy deficit participants reported.^44^ Results demonstrated that the actual weight loss led by this reported energy deficit was less than predicted, indicating that participants may reported excessive daily energy reduction.

**Table 4:** Subgroup analysis results based on energy prescription

	Energy prescribed		Not prescribed	
	*N*_*I*_*/N*_*c*_	*MD* with 95%CI	*N*_*I*_*/N*_*c*_	*MD* with 95%CI
**Primary Outcomes**				

WL (%)	387/389	−1.25 (−1.74, −0.75)	232/223	−3.08 (−3.42, −2.73)
WC (cm)	267/270	−2.05 (−3.38, −0.72)	77/73	−3.11(-4.53, −1.69)
BMI (kg/m^2^)	254/257	−0.44(-0.58, −0.30)	157/151	−1.14(-1.30, −0.98)
FM(kg)	256/255	−0.91(-1.41, −0.42)	172/163	−1.64(-2.19, −1.09)
BLM (kg)	261/264	−0.11(-0.57, 0.35)	198/191	−0.68 (−1.14, −0.23)
VF (kg)	75/78	−0.03 (−0.15, 0.10)	70/67	−0.06 (−0.14, 0.02)

**Secondary Outcomes**				

EI (kcal)	328/331	−51.53 (−106.62, 3.55)	174/169	−445.96 (−468.58, −423.33)

**Exploratory Outcomes**				

SBP (%)	201/205	−0.58 (−3.20, 2.03)	150/143	−3.44 (−5.83, −1.05)
DBP (%)	201/205	−2.11 (−6.65, 2.44)	150/143	−0.80 (−3.63, 2.03)
HR (bpm)	114/115	−0.39 (−3.07, 2.28)	114/109	0.76 (−1.11, 2.63)
TG (mg/dL)	299/302	0.84 (−4.03, 5.71)	199/193	−9.76 (−16.80, −2.72)
TC (mg/dL)	299/302	1.37 (−2.93, 5.66)	149/146	1.69 (−5.36, 8.74)
HDL (mg/dL)	299/302	1.18 (−0.04, 2.39)	210/203	−0.06 (−1.40, 1.27)
LDL (mg/dL)	274/278	1.38 (−2.36, 5.12)	199/193	2.18 (−2.18, 6.55)
FG (mg/dL)	338/341	−1.31 (−3.63, 1.00)	199/193	−3.50 (−7.03, 0.02)
FI (μIU/mL)	151/154	0.61 (−1.28, 2.49)	210/203	−2.72 (−4.39, −1.04)
HOMA-IR	220/224	−0.02 (−0.52, 0.48)	160/155	−0.42 (−0.56, −0.29)
HbA1c (%)	77/78	0.00 (−0.20, 0.19)	187/180	−0.13 (−0.37, 0.11)
RMR (kcal)	111/110	19.85 (−2.01, 41.72)	25/25	15.00 (−68.79, 98.79)

WL, weight loss; EI, energy intake; WC, waist circumference; BMI, body mass index; FM, fat mass; BLM, lean body mass; VF, visceral fat; SBP, systolic blood pressure; DBP, diastolic blood pressure; HR, heart rate; TG, triglycerides; TC, total cholesterol; HDL, high-density cholesterol; LDL, low-density cholesterol; FG, fasting glucose; FI, fasting insulin; HOMA-IR, Homeostasis Model Assessment-Insulin Resistance; HbA1c, Hemoglobin A1c; RMR, resting metabolic rate.

In anthropometric parameter and body composition analysis, the subgroup analysis showed more decreases in waist circumference, body mass index, and fat mass in the *ad libitum* intake condition than in the isocaloric condition. No difference was found in visceral fat between the two subgroups. Lean body mass in the TRE group with *ad libitum* intake was effectively reduced compared to the control group (−0.68 kg, 95%CI: −1.14 to −0.23), while in studies with isocaloric intake, there was no difference between the two groups (−0.11 kg, 95%CI: −0.57 to 0.35). In metabolic risk factors, systolic blood pressure (−3.44%, 95%CI: −5.83 to −1.05), triglycerides (−9.76 mg/dL, 95%CI: −16.80 to −2.72), fasting insulin (−2.72 μIU/mL, 95%CI: −4.39 to −1.04), and HOMA-IR (−0.42, 95%CI: −0.56 to −0.29) decreased more in studies with *ad libitum* intake but changed less once energy intake was controlled.

In conclusion, TRE demonstrated improvements in weight loss, anthropometric parameters, body composition, and metabolic health measurements. Subgroup analysis results based on energy prescription support the hypothesis that energy restriction contributed to these benefits, as body weight and metabolic parameters improved less once energy intake was controlled.

### Eating time of day also improves the health status of patients on TRE diets

The subgroup analysis described above demonstrated the role of energy deficits in improving health outcomes in TRE regimes, yet improvements in weight loss percentage, anthropometric parameters, body composition, and some metabolic indicators still existed under the isocaloric intake condition. Since many studies have confirmed the significance of eating time of day in the TRE diet, we conducted further subgroup analysis by taking the time of day into consideration, categorizing four subtypes as e-TRE and d-TRE, with and without energy prescription, to separate the effect of eating time of day from energy consumption (Table 5). We investigated whether the alignment with eating time of day could lead to benefits under the *ad libitum* condition (energy not prescribed) and, if so, whether these benefits still exist once the amount of energy was controlled (energy prescribed).

**Table 5:** The results of subgroup analysis based on energy prescription and eating time of day

	Energy Prescribed				Not prescribed			
	e-TRE		d-TRE		e-TRE		d-TRE	
	*N*_*I*_*/N*_*C*_	*MD* with 95%CI	*N*_*I*_*/N*_*C*_	*MD* with 95%CI	*N*_*I*_*/N*_*C*_	*MD* with 95%CI	*N*_*I*_*/N*_*C*_	*MD* with 95%CI

**Primary Outcomes**								

WL (%)	192/193	−1.22 (−2.06, −0.37)	28/27	−0.20 (−1.84, 1.44)	21/19	−4.23 (−6.96, −1.50)	117/113	−3.26 (−3.44, −3.09)
WC (cm)	114/115	−1.53 (−3.19, 0.12)	12/11	0.40 (−6.96, 7.76)	21/19	−4.20 (−6.22, −2.18)	56/54	−2.29 (−4.09, −0.50)
BMI (kg/m2)	85/86	−0.34 (−0.88, 0.21)	28/27	−0.14 (−0.72, 0.44)	21/19	−1.10 (−1.43, −0.77)	53/49	−1.14 (−1.70, −0.59)
FM (kg)	130/131	−0.83 (−1.81, 0.15)	28/27	−0.59 (−1.80, 0.63)	21/19	−1.70 (−2.49, −0.91)	117/112	−1.62 (−2.33, −0.91)
BLM (kg)	130/131	−0.27 (−0.75, 0.22)	28/27	0.30 (−1.15, 1.76)	21/19	−1.60 (−2.23, −0.97)	117/112	−0.56 (−1.10, −0.03)
VF (kg)	192/193	−1.22 (−2.06, −0.37)	28/27	−0.20 (−1.84, 1.44)	21/19	−4.23 (−6.96, −1.50)	117/113	−3.26 (−3.44, −3.09)

**Secondary Outcomes**								

EI (kcal)	130/131	−85.18(-209.82, 39.46)	16/16	−27 (−506.29, 452.29)	21/19	−442 (−629.02, −254.98)	70/67	−428.77 (−468.44, −389.11)

**Exploratory Outcomes**								

SBP (%)	114/115	−1.76 (−5.23, 1.71)	–	–	21/19	−3.75 (−6.77, −0.73)	95/92	−3.16 (−6.53, 0.21)
DBP (%)	114/115	−3.31 (−6.53, −0.10)	–	–	21/19	−0.84 (−9.09, 7.41)	95/92	−0.84 (−4.87, 3.19)
HR (bpm)	114/115	−0.39 (−3.07, 2.28)			21/19	2.10 (−2.24, 6.44)	70/67	0.44 (−1.90, 2.77)
TG (mg/dL)	130/131	3.29 (−7.63, 14.21)	28/27	−14.65 (−79.5, 50.15)	21/19	2.70 (−15.31, 20.71)	84/82	−5.85 (−13.81, 2.11)
TC (mg/dL)	130/131	4.32 (−3.27, 11.92)	28/27	−0.15 (−29.74, 29.45)	21/19	6.00 (−2.44, 14.44)	45/44	3.02 (−9.70, 15.74)
HDL (mg/dL)	130/131	1.80 (−0.62, 4.23)	28/27	0.52 (−4.27, 5.31)	21/19	0.10 (−2.02, 2.22)	95/92	−0.64 (−1.81, 0.53)
LDL (mg/dL)	130/131	2.53 (−3.68, 8.74)	28/27	10.80 (−3.51, 25.11)	21/19	6.70 (1.53, 11.87)	84/82	2.71 (−2.20, 7.61)
FG (mg/dL)	130/131	−0.80 (−3.90 2.30)	28/27	−1.42 (−8.18, 5.34)	21/19	−1.00 (−3.91, 1.91)	84/82	−3.42 (−7.12, 0.29)
FI (μIU/mL)	61/61	−3.21 (−8.06, 1.64)	28/27	1.66 (−2.55, 5.86)	21/19	−3.80 (−6.04, −1.56)	95/92	−3.07 (−5.54, −0.60)
HOMA-IR	130/131	−0.57 (−1.30, 0.16)	28/27	0.15 (−0.73, 1.03)	21/19	−0.90 (−1.46, −0.34)	45/44	−0.32 (−0.73, 0.09)
HbA1c (%)	45/45	0.00 (−0.28, 0.28)	–	–	21/19	−0.20 (−0.27, −0.13)	95/92	−0.02 (−0.15, 0.11)
RMR (kcal)	16/16	62.30 (−57.45, 182.05)	16/16	31.60(-79.52, 142.72)	–	–	25/25	15.00 (−68.79, 98.79)

WL, weight loss; EI, energy intake; WC, waist circumference; BMI, body mass index; FM, fat mass; BLM, lean body mass; VF, visceral fat; SBP, systolic blood pressure; DBP, diastolic blood pressure; HR, heart rate; TG, triglycerides; TC, total cholesterol; HDL, high-density cholesterol; LDL, low-density cholesterol; FG, fasting glucose; FI, fasting insulin; HOMA-IR, Homeostasis Model Assessment-Insulin Resistance; HbA1c, Hemoglobin A1c; RMR, resting metabolic rate.

Weight loss was greater in e-TRE than in d-TRE under the *ad libitum* condition, and decreased in e-TRE but not in d-TRE under the isocaloric condition. Energy intake reduced more in e-TRE than in d-TRE under the *ad libitum* condition but did not differ under the isocaloric condition. Waist circumference, fat mass, lean body mass, fasting insulin levels, and HbA1c levels showed greater improvements in e-TRE than in d-TRE without energy prescription but did not differ once the amount of energy was controlled. HOMA-IR improved only in e-TRE when the energy amount was not prescribed. These results demonstrated that for those health outcomes that improved under the *ad libitum* condition, the alignment with the time of day could, to some extent, strengthen the utility of TRE.

## Discussion

The current systematic review and meta-analysis explored the effect of TRE on weight loss and metabolic health in individuals with overweight and obesity statuses based on randomized controlled trials involving 19 studies with 22 intervention arms and 1201 participants. To our knowledge, this was the first systematic review and meta-analysis investigating the reason why TRE leads to weight loss and improves metabolism. The data indicated that TRE was effective in weight loss and metabolic health, and these benefits were due to a joint effect of energy restriction and eating time of day. Subgroup analysis of four subgroups further indicated that energy restriction plays a dominant role compared to the eating time of day. In the isocaloric condition, only e-TRE improved health outcomes while d-TRE with *ad libitum* intake was also effective, with considerable energy reduction, it was not as beneficial as e-TRE.

### Weight loss and energy intake in TRE strategies

TRE reduced body weight and actual energy consumption but did not achieve a clinical significance of 5% weight loss. This modest reduction was in line with previous meta-analysis so we further do meta-regression to explore the mechanism of weight loss in TRE strategies.^34^^,^^35^^,^^36^^,^^45^ Meta-regression showed that weight loss was significantly related to energy consumption (*Z* = 5.23, p < 0.001) and the eating window (*Z* = 2.26, p = 0.024) but was not correlated with the intervention duration (*Z* = 0.36, p = 0.72). This demonstrated an increased capacity for TRE to cause weight loss by a greater calorie deficit and shorter eating window within a safe range.^46^ Restricting eating window can reduce eating window intentionally or unintentionally and achieve weight loss. Since most people have a daily eating window that exceeds 12 h,^19^^,^^47^^,^^48^^,^^49^ previous studies reported 8 h to be a safe and adherable eating period, and an excessively restricted eating window might ironically result in an increased risk of binge eating. The absence of a correlation between weight loss and intervention duration suggested that more studies with longer and diverse intervention durations are needed to explore the ambiguous effects of intervention duration on TRE outcomes. Some research suggested that the threshold for the effect of TRE strategies was around 12 weeks when the intervention effect was likely to be the best with the highest dietary adherence, after which the effectiveness might decrease as the adherence rate lowered.^11^^,^^50^ Therefore, the relevance of energy balance, the eating window, and body weight need to be rigorously assessed, and more objective measurements of daily energy intake and compliance are needed.^51^ Although the weight loss magnitude was modest, subgroup analysis indeed showed the advantage of eating time-of-day in TRE. For people with metabolic symptoms, TRE can also be used as a strategy to improve their metabolic indicators.

Subgroup analysis indicated more weight loss under the *ad libitum* intake condition than the isocaloric condition. Moreover, both e-TRE and d-TRE reduced body weight under the *ad libitum* condition with substantial energy deficit (approximately ∼450 kcal/d), but e-TRE was more effective, and the weight loss benefits of d-TRE disappeared when the energy intake was actually the same (isocaloric condition). The weight loss of e-TRE under prescribed conditions suggested that, to some extent, TRE could indeed improve the health status of people with overweight and obesity statuses through appropriate time-of-day eating in the absence of energy reductions. In conclusion, it is possible that weight loss in the TRE strategy might have been driven by the combination of energy deficit and eating time of day. However, energy reduction had a more robust effect.

### TRE improves anthropometric parameters, body composition, and some metabolic outcomes

In the analysis of anthropometric parameters and body composition, TRE reduced waist circumference, BMI, fat mass, and lean body mass but did not affect visceral fat. From this perspective, TRE may not improve fat mass loss while maintaining lean body mass compared to traditional calorie-restricted diets, where weight loss is always accompanied by a concomitant reduction in lean body mass.^4^^,^^48^^,^^52^ The decrease in lean mass may have been due to the inclusion of individuals with overweight and obesity statuses in this meta-analysis, and physical exercise with adequate protein intake can regulate the loss of lean tissue.^53^ The different changes in lean body mass under isocaloric and *ad libitum* conditions in the subgroup analysis illustrate the importance of energy restriction in sustaining lean body mass.

In metabolic risk factors, TRE improved systolic blood pressure and glycemic (fasting glucose, fasting insulin, and HbA1c levels) indicators, while other parameters, including diastolic blood pressure, triglyceride, total cholesterol, high-density cholesterol, low-density cholesterol levels, HOMA-IR, and resting metabolic rate did not change between the TRE and control groups. Blood pressure is related to metabolic disease and heart failure, but the results of studies on the impact of TRE on blood pressure have been mixed. This meta-analysis found decreased systolic blood pressure in the TRE group under the *ad libitum* intake condition with sizable energy reduction, whereas diastolic blood pressure remained the same, consistent with another recently published meta-analysis.^45^^,^^54^ A study in shift workers found a significant decrease in blood pressure among participants with elevated systolic blood pressure (≥130 mmHg) or diastolic blood pressure (≥85 mmHg) compared to those with normal initial levels, indicating the salience of TRE in people with elevated cardiometabolic risks at baseline.^55^ In addition, subgroup analysis showed that systolic blood pressure was decreased under the *ad libitum* condition and that e-TRE was more effective compared to d-TRE only with relatively lower energy intake.

Plasma lipids are another factor affecting metabolic disease as well as cardiovascular disease. However, the benefits of TRE are more salient in people with metabolic syndrome,^39^ resulting in non-significant improvements in triglycerides, high-density cholesterol, and low-density cholesterol levels in this meta-analysis where most of the participants were not patients with metabolic symptoms. Besides improved triglycerides under the *ad libitum* condition, lipid levels between the isocaloric and *ad libitum* subgroups were not different. In contrast to the utility of TRE in people with metabolic syndrome, TRE showed no improvement in lipid levels in this meta-analysis, suggesting that TRE may act more as anti-hypertensive therapy in those with elevated baseline lipid levels.^56^ Glycemic levels and insulin resistance are factors that contribute to both metabolic disease and cardiovascular disease. Previous clinical data and this meta-analysis demonstrated a robust effect of TRE on fasting glucose and fasting insulin levels, indicating TRE as a useful treatment for type 2 diabetes, where glucose management is critical to minimizing diabetes-associated complications and improving health and the quality of life.^57^^,^^58^

Consistent with the conclusions of some previous studies that e-TRE was superior to d-TRE in terms of metabolic improvement, this meta-analysis found similar results that body composition and metabolic parameters in e-TRE were better than those in d-TRE under the *ad libitum* intake condition, while d-TRE showed no benefits once energy intake was controlled. In other words, the advantages of energy deficits for weight loss and metabolism were more salient than those of the eating time of day in TRE strategies. Individuals who consumed energy amounts over baseline levels could gain weight even using the TRE strategy characterized by the time of day. Poor diet quality, such as low-nutrient, high-fat foods, could also limit the effectiveness of TRE.^12^ The estimate of most outcomes in this meta-analysis did not reach clinical significance. However, the intervention effect of TRE in health improvement was greatest in the e-TRE subgroup with considerable energy deficit. The relatively modest magnitude has been demonstrated in some previous meta-analysis, some attributed this to the short intervention duration^45^ while other did not find the association between health improvement and duration.^34^ Thus, more long-term TRE studies are needed to get a more solid conclusion, both in people with overweight or obesity status and patients with metabolic symptoms.

In conclusion, TRE could effectively lead to modest weight loss, decreases in waist circumference, body mass index, fat mass, and lean body mass, and improvements in systolic blood pressure, fasting glucose, fasting insulin, and HbA1c levels relative to the control group. Yet, TRE did not impact visceral fat, diastolic blood pressure, triglyceride, total cholesterol, high-density cholesterol, low-density cholesterol levels, HOMA-IR, or resting metabolic rate. Subgroup analysis based on energy intake and eating time of day suggested that energy restriction and eating time of day collectively led to weight loss and improved metabolic health in the TRE diet strategy, but the effect of energy reduction was more vital.

### Limitations of the study and future TRE research

A limitation of this systematic review and meta-analysis was that several related outcomes were not examined due to a small number of trials, and the intervention strategies were relatively simple. Except for one study by Liu et al.^52^ with an intervention duration of one year (48 weeks), the remaining studies had relatively short intervention durations. Most were 8 and 12 weeks and lacked follow-up on the long-term effects of TRE strategies. Some studies^11^^,^^51^ suggested that the intervention effect of TRE showed an inverted U-shaped curve that increased initially and then decreased with the intervention duration. However, in this meta-analysis, we were not able to perform a nonlinear meta-regression to test this hypothesis due to the limitations of the intervention duration lengths in the included studies. Second, subgroup analysis and meta-regression using group-level data suffer from ecological fallacy. Therefore, the conclusion does not lead to a causal relationship and cannot be generalized to the individual level.^59^^,^^60^ That is, the intervention strategies demonstrated to be effective in this meta-analysis are not necessarily helpful to a certain person with overweight or obesity status. Moreover, given that there was only one study, that by Zhang et al.,^21^ under e-TRE without energy prescription in the subgroup analysis, great caution must be taken when describing the interaction of energy consumption and eating time of day on TRE for weight loss and metabolic improvement.^61^ Third, we did not distinguish patients with metabolic symptoms from other participants as the number of RCT studies in those patients was small, yet previous studies reported TRE to be a more efficient dietary strategy for people with metabolic syndrome.^56^ Forth, we did not include Ramadan fasting in this meta-analysis because the restriction of eating time-of-day after sunset and at night did not apply to the majority of non-Muslim population. Given the relative youth of the body of literature examining TRE in humans, future studies should attempt to identify and empirically test the influence of multiple different intervention approaches on TRE for weight loss and improvements in metabolic parameters, including what, when, and how much individuals eat daily. Fifth, the adequacy of energy intake measures was not considered in the exclusion criteria when screening the articles.

Although most clinical RCTs measured adherence during TRE, only four of them reported adherence quantitatively as the percentage of the participating days or the number of adherent days per week. Moreover, self-reported measures of adherence, such as remote video by Skype or daily adherence logs, rely on participants’ honesty and may be affected by social desirability bias.^11^^,^^62^ In addition to using adherence to predict long-term weight maintenance, the distribution of protein and carbohydrate intake is crucial for understanding the full benefit of TRE as these macronutrients are vital to modifying body composition and glycemic levels.^63^^,^^64^ Biological hunger and satiety can result in urges to eat and are related to TRE eating time. However, few studies have measured leptin levels or reported subjective hunger. A recent study proposed that participants consuming a morning-loaded diet reported significantly lower hunger and reduced caloric intake at lunch and evening, thus contributing to enhanced weight loss.^65^ Additional research is needed to examine the fluctuation of hunger levels throughout the day and its association with eating window time. Gender is also a physiological factor that could affect TRE outcomes. Most participants in this meta-analysis were female (65.07%), whereas male participants accounted for a large proportion only in studies of individuals participating in physical activities or special populations, such as firefighters or athletes. Thus, more studies are needed to examine whether gender differences have an impact on TRE strategy with the same baseline body composition and metabolic risk factors.

Some public social issues should also be considered when promoting the TRE strategy to a broader range of people. The disrupted activity-rest cycle caused by artificial light and industrialization indirectly disrupts the natural daily cycle of feeding and fasting and facilitates excessive caloric intake.^7^ Additionally, it is difficult for shift workers, people with low economic levels, and those who lack social support to restrain their eating window to less than 12 h a day.^43^^,^^66^ Therefore, the scope of future studies should be expanded to understand the feasibility of TRE in these populations.

## STAR★Methods

### Key resources table

**Table:** 

REAGENT or RESOURCE	SOURCE	IDENTIFIER
**Deposited data**		

Mendeley Data	This article	https://data.mendeley.com/preview/vs4td5yy94?a=1092a21d-f86b-4dcd-9bbb-91142b7cd13c

**Software and algorithms**		

R 4.3.0	R project	https://www.r-project.org/
RStudio 2023.03.0 + 386	RStudio	https://posit.co/download/rstudio-desktop/
Body Weight Planner	National Institutes of Health	www.niddk.nih.gov/bwp

### Resource availability

#### Lead contact

Further information and requests for resources should be directly to and will be fulfilled by the lead contact, Guojie Ma (magj@snnu.edu.cn).

#### Materials availability

This study did not generate unique reagents.

#### Data and code availability

• The summary statistics of the present meta-analysis have been deposited to the Mendeley at https://data.mendeley.com/preview/vs4td5yy94?a=1092a21d-f86b-4dcd-9bbb-91142b7cd13c and accession numbers are listed in the key resources table.
• All original code is also available at the Mendeley Data https://data.mendeley.com/preview/vs4td5yy94?a=1092a21d-f86b-4dcd-9bbb-91142b7cd13c.
• Any additional information required to reanalyze the data reported in this work is available from the lead contact upon request.

### Experimental model and study participant details

Nineteen studies with a total of 1201 participants were included in the meta-analysis. The mean age of participants ranged from 22.7 to 65 years, and the mean BMI ranged from 27.8 to 38.9 kg/m^2^. Females accounted for 65.07% of the included participants. Statistics related to the participants in these nineteen studies are summarized in Table 1. Only five of the included studies^20^^,^^22^^,^^23^^,^^48^^,^^67^ reported the race or ethnicity of the participants, so we were unable to conclude the race or ethnicity information for the participants. The effect of gender on the results of the meta-analysis was not specifically measured. We included participants’ data collected by Lowe et al.^20^ only using the in-person cohort method in the meta-analysis, as only these data was reported quantitatively.

### Method details

#### Data extraction and pooling methods

As the outcomes are continuous variables and can be converted to uniform units, we used mean difference (*MD*) as the pooled statistic to reflect the combined effect. The random-effects model was carried out to estimate the pooled effect size and visualized all results through the forest plots. The number of participants (*N*), the mean difference (*M*), and the standard deviation (*SD*_diff_) of the outcomes from baseline to endpoint for the intervention and control groups were presented as the final data to be processed in R. Below formula was used to calculate the standard deviation of difference between the intervention and control groups (*SD*_diff_):

$${SD}_{\text{diff}}=\sqrt{{SD}_{baseline}^{2}+{SD}_{endpoint}^{2}-\left(2\times Corr\times {SD}_{baseline}\times {SD}_{endpoint}\right)}$$

Note: *SD*_baseline_ is the standard deviation of intervention/control group at baseline, and *SD*_endpoint_ is the standard deviation of intervention/control group at endpoint. When the standard deviation was not directly reported in the original article, it would be calculated as *SD* = $\sqrt{N}\left(UCI-LCI\right)/2t_{0.05}$ or as *SD* = *SE* $\sqrt{N}$ depending on whether the statistics reported in the original article was a 95% confidence interval (95% CI) or a standard error (*SE*).

For those studies that initially reported the standard deviations of difference between the intervention and control (SD_*diff*_) groups, we imputed the correlation coefficients directly through this formulate in both the intervention and control group:

$$\text{Corr}=\frac{{SD}_{baseline}^{2}+{SD}_{endpoint}^{2}-{SD}_{diff}^{2}}{2\times {SD}_{baseline}\times {SD}_{endpoint}}$$

The average of correlation coefficient (${Corr}_{average}$) in those studies was used as the final correlation coefficient in meta-analysis to calculated the SD_*diff*_ for the intervention and control group in the studies that did not initially report them.

$${SD}_{diff}=\sqrt{{SD}_{baseline}^{2}+{SD}_{endpoint}^{2}-\left(2\times {Corr}_{average}\times {SD}_{baseline}\times {SD}_{endpoint}\right)}$$

We use a simple approach to extract data from the two-periods, two-intervention crossover studies. Since the individual participant data are not available, and neither carry-over nor period effects are thought to be a problem, we take all measurements from intervention group during both stages and all measurements from control group during both stages, analyzing the crossover trial outcomes as RCT trials.^43^

#### Body weight planner software

The Body Weight Planner Software (www.niddk.nih.gov/bwp) can be used to predicted the energy deficit required to reduce body weight from the initial state to the goal state. The initial body weight was set as the mean body weight at baseline under *ad libitum* condition. The age and number of days needed to reach goal weight was calculated as mean age and mean intervention durations based on the data in Table 2. Height was imputed using mean weight and BMI reported at baseline, and gender was set to female considering most of participants (65.07%) included in these articles were female. Physical activity in work and life was set as very light and light.

### Quantification and statistical analysis

#### Clinical research

Articles for this meta-analysis were searched through four databases, PubMed, Embase, Scopus, and the Cochrane library from inception to 7 September, 2022 with no language restriction. We searched for studies by title, abstract, and keyword. Intervention terms included “time-restricted” or “time restriction”, and participants terms included “overweight” or “obese”. For detailed searched strategies see Table S2. The included studies’ reference lists, relevant systematic reviews and meta-analysis were also manually screened to identify other potentially eligible studies. Primary outcomes include weight loss percentage,^42^ waist circumference, and body composition change. Secondary outcome was change in energy intake to explain the primary outcomes. Other exploratory outcomes contained blood pressure (systolic and diastolic blood pressure), blood glucose and lipids levels (fasting glucose levels, fasting insulin levels, insulin sensitivity through HOMA-IR, total cholesterol, high-density cholesterol, low-density cholesterol, HbA1c levels) to reflect changes in other metabolic health.

#### Article selecting criteria

Exclusive criteria: studies of TRE or fasting less than 12 h, active exercise people, intervention duration of fewer than 4 weeks, and religious fasting such as Ramadan since it is a specific fasting pattern which allowed people to drink or eat only after sunset and fast during daylight, and is less frequently used by non-Muslim people with overweight and obesity.^68^

Inclusive criteria: Adults with a BMI≥25 kg/m^2^ were included even though some participants have metabolic syndrome, prediabetes, or type 2 diabetes.

#### Meta-analyses

Meta-analysis was conducted using “meta” and “metafor” function packages in RStudio (x64 4.0.3). Clinical heterogeneity was assessed by using the I-squared statistic, and the level of heterogeneity between these studies was considered high when *I*^*2*^ > 50% or p < 0.05. Subgroup analysis was conducted based on the type of intervention (i.e., isocaloric or eat *ad libitum*) and eating time-of-day (i.e., e-TRE or d-TRE), and meta-regression was carried out to test the effect of the eating window and intervention duration on weight loss percentage and energy intake. In addition, funnel plots and Egger’s test were used to assess the publication bias when at least ten studies were included in a meta-analysis. When p < 0.05, we considered there was a publication bias to the result.

#### Additional resources

We conducted this meta-analysis according to the Preferred Reporting Items of Systematic Reviews and Meta-analysis (PRISMA) 2020 guidelines (Table S1). The protocol for this meta-analysis was registered at the International Prospective Register of Systematic Reviews (CRD42022380696).

Our study has not generated or contributed to a new website and it is not part of a clinical trial.
