# Supplementary material for: Time-restricted eating improves health because of energy deficit and circadian rhythm: A systematic review and meta-analysis
Source: iScience. 2024 Jan 26;27(2):109000. doi: 10.1016/j.isci.2024.109000 (PMC10865403; doi:10.1016/j.isci.2024.109000)
Supplement: Document S1. Figures S1 and Tables S1–S6 [file mmc1.pdf]

iScience, Volume *27*

Supplemental information

Time-restricted eating improves health because of energy deficit and circadian rhythm: A systematic review and meta-analysis

Yuwen Chang, Tingting Du, Xiangling Zhuang, and Guojie Ma

Supplemental Figures

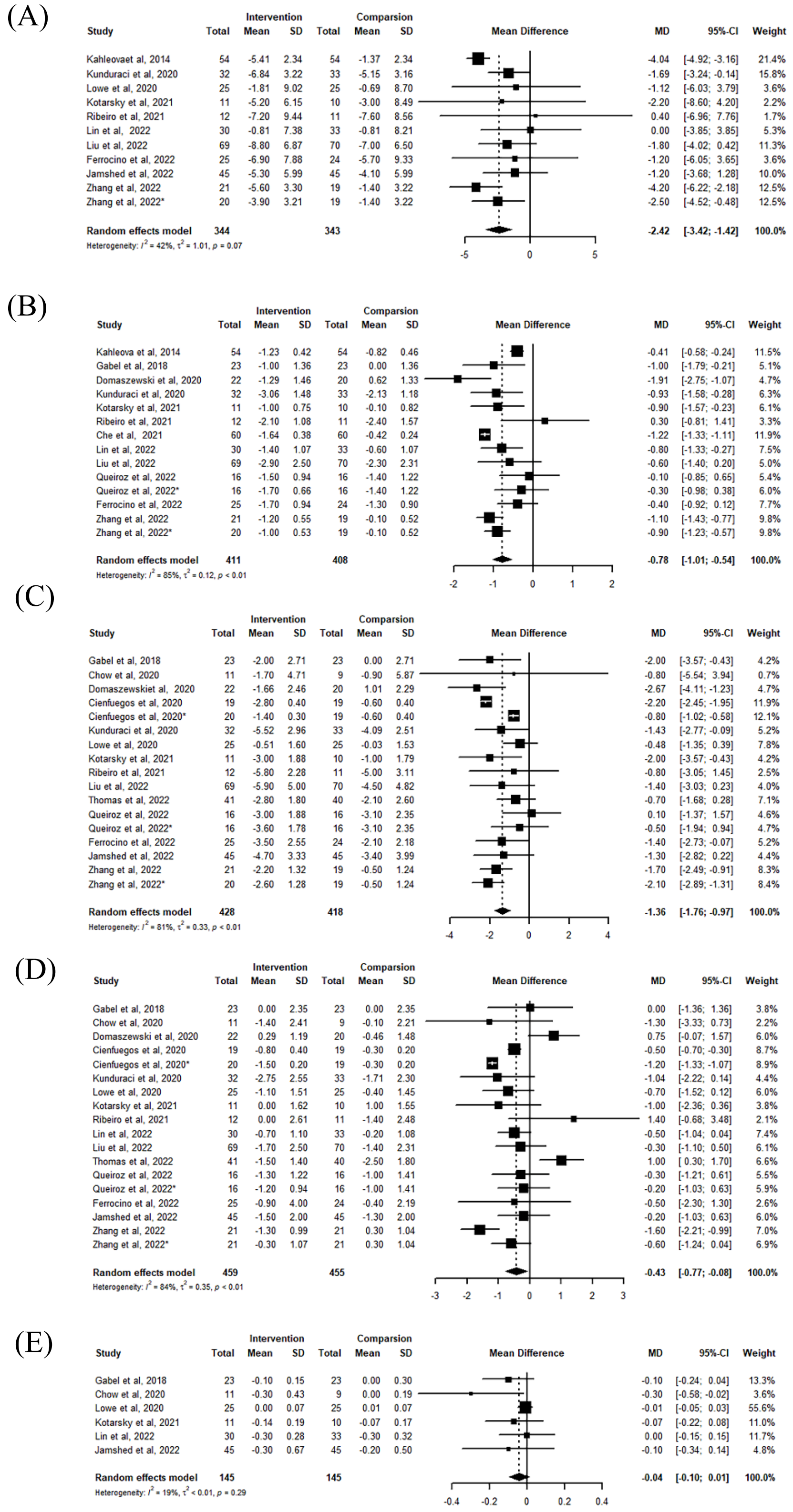

Figure S1. The effect of TRE on anthropometric parameter and body composition. Related to Table 3. We present the forest plots of primary outcomes. (A) waist circumference (cm), (B) body mass index (kg/m^2), (C) fat mass (kg), (D) body lean mass (kg), (E) visceral fat (kg).

Supplemental Tables

Table S1. The PRASMA Checklist. Related to Figure 1, 2, 3 and Table 2, 3, 4, 5.

SECTION/TOPIC	#	CHECKLIST ITEM	REPORTED ON PAGE #
**TITLE**			
Title	**1**	Time-restricted eating improves health because of energy deficit and circadian rhythm: A systematic review and meta-analysis	1
**ABSTRACT**			
Structed summary	**2**	background; objectives; data sources; study eligibility criteria, participants, and interventions; study appraisal and synthesis methods; results; limitations; conclusions and implications of key findings; systematic review registration number	NA
**INTRODUCTION**			
Rational	**3**	Time restricted eating (TRE) can lead to weight loss and some metabolic health outcomes.	1-2
Objectives	**4**	But to what extent are these benefits caused by energy restriction or eating time-of-day is unclear. Meta-analysis on this topic about patients with overweight or obesity undergoing TRE more than 4 weeks have not published yet.	2-3
**METHODS**			
Protocol and registration	**5**	https://www.crd.york.ac.uk/PROSPERO/display_record.php?RecordID=380696	18
Eligibility criteria	**6**	Participants: Adults with BMI $\geq$ 25 kg/m^2<br><br>Intervention：TRE with fasting time more than 12 hours and intervention duration more 4 weeks<br><br>Comparison: Normal diet with fasting time less than 12 hours<br><br>Outcomes: Primary outcomes are weight loss percentage, waist circumference and body compositions, secondary outcomes are energy intake, exploratory outcomes are metabolic change	18
Information sources	**7**	through four databases, PubMed, Embase, Scopus, and the Cochrane library from inception to 7 September, 2022 with no language restriction	17-18
Search	**8**	PubMed: (((time restriction OR time restricted) AND (eating OR feeding) [Title/Abstract])) AND (overweight OR weight loss OR obese OR obesity [Title/Abstract])	Supplemental information Table S2
Study selection	**9**	Screen the title and abstract, then the full article. The included studies' reference lists and relevant systematic reviews and meta-analysis were also manually screened.	Figure 1
Data collection process	**10**	Two authors collect the data independently and any inconsistency is discussed with another author.	13

Risk of bias across studies	**11**	Assess by the Cochrane Collaboration Risk of Bias 2.0 Tool for RCTs and Risk of Bias 2.0 for crossover trials.	Supplemental information Table S4-5.
Summary measures	**12**	Difference in means (MD) between the intervention and control groups from baseline to endpoint	16-17
Synthesis of results	**13**	Access clinical heterogeneity by using the I-square statistic	16-17
Additional analyses	**14**	Subgroup analysis based on whether the energy is prescribed and the early and delay eating time-of-day. Meta-regression about the effect of eating window and intervention duration on weight loss percentage and actual energy intake outcomes. GRADE to assess the quality of evidence.	6-8
**RESULTS**			
Study selection	**15**	3156 studies were identified and 19 studies entered the final meta-analysis	Figure 1
Risk of bias across studies	**16**	In included studies, 7 were high of risk bias, 3 were of some concern, 9 were low of risk bias.	Supplemental information Table S4-5.
Results of individual studies	**17**	TRE improved WL%, WC, BMI, FM, BLM, EI, SBP, FG, FI, and HbA1c%. TRE did not change VF, DBP, TG, TC, HDL, LDL, HOMA-IR, and RMR.	5-6
Additional analysis	**18**	Subgroup analysis indicate both energy reduction and eating time-of-day contribute to the health improvement, yet the effect of energy reduction is more obvious. Meta-regression suggests that eating window and actual energy intake related to the weight loss significant, intervention duration is not associate with neither weight loss nor energy intake.	6-10
**DISCUSSION**			
Summary of evidence	**19**	Both energy reduction and eating time-of-day contribute to the health improvement, yet the effect of energy reduction is more obvious.	8-10
limitations	**20**	We did not exclude some people with metabolic symptoms from other participants	10-11
Conclusion	**21**	TRE can effectively lead to modest WL, reduced WC, BMI, FM, and BLM, as well as improved SBP, FG, FI, and HbA1c relative to the control group. Yet, TRE did not impact VF, DBP, TG, TC, HDL, LDL, HOMA-IR, and RMR. Subgroup analysis based on energy intake and eating time-of-day suggests that it is the energy restriction and eating time-of-day that collectively leads to weight loss and metabolic health in the TRE diet strategy, yet the effect of energy reduction is more vital.	11

We conducted this meta-analysis according to the Preferred Reporting Items of Systematic Reviews and Meta-analysis (PRISMA) 2020 guidelines.

Table S2. Search strategies for each database. Related to the STAR Methods

Database	Search strategy
PubMed	(((time restriction OR time restricted) AND (eating OR feeding) [Title/Abstract])) AND (overweight OR weight loss OR obese OR obesity [Title/Abstract])
Scopus	(TITLE-ABS-KEY ("time restrict*" AND (eating OR feeding)) AND TITLE-ABS-KEY (obesity OR obese OR overweight))
Embase	'time restrict*' AND ('feeding'/exp OR feeding OR 'eating'/exp OR eating) AND (obese OR obesity OR overweight)
The Cochrane library	(time restrict* AND (eating OR feeding)) AND (obesity OR obes* OR overweight) [Title/Abstract/Keywords]

Articles for this meta-analysis were searched through four databases, PubMed, Embase, Scopus, and the Cochrane library from inception to 7 September, 2022 with no language restriction. We searched for studies by title, abstract, and keyword.

Table S3. Detailed information about TRE intervention. Related to Table 2.

First Author With Year	Country	Macronutrient	Consultation	Exercise	Adherence
**Parellel-group studies**					
Gabel et al, 2018	USA	Not report	15 min instruction on how to complete the food records at baseline	keep their habitual physical activity	a daily adherence log
Chow et al, 2020	USA	No additional instruction	No additional instruction	Not report	document all oral intake using the mCC application
Domaszewski et al, 2020	Poland	Not report	Not report	keep their habitual activity	Not report
Cienfuegos et al, 2020	USA	Not report	Not report	Keep habitual physical activity	A daily adherence log and meet with coordinator weekly
Kunduraci et al, 2020	Turkey	determined personally	clinical dietician whenever they wanted	keep habitual activity	subjects were contacted once a week via telephone, and face to face for four-week periods
Lowe et al, 2020	USA	No recommendation	Daily reminders about eating window	no recommendation	through the app
Peeke et al, 2021	USA	25-35% fat, 45-55% carbohydrate 20-30% protein	Weekly safety and tolerability assessments	average daily step between 7000 to 10,000 each day	attendance on phone/video calls and providing study measurements
Kotarsky et al, 2021	USA	Not strictly controlled	A specialist in sprots dietetics provide all dietary instructions	aerobic exercise and supervised resistance training	dietary intake and adherence were measured using Food Processor software
Ribeiro et al, 2021	Brazil	45% carbohydrate, 20-27% protein, 26-35% fat	food plan and support by registered dietitian	1 hour training weekly.	not report
Che et al, 2021	China	Not report	Met weekly with the research supervisor	keep their habitual physical activity	record the eating window through a daily log
Lin et al, 2022	China	Not report	Provide information and encouragement	eight 30-min exercise sessions	record daily food intake and sent them to the dietitian instantly for a better record
Liu et al, 2022	China	40 to 55% carbohydrates, 15 to 20% protein, 20 to 30% fat	dietary counseling by trained health coaches	not report	follow-up telephone or app messages twice per week with a health coach every two weeks
Thomas et al, 2022	USA	not report	Met weekly during the first 12 weeks and then monthly.	150 min/wk of moderate-intensity activity	self-reported adherence and photographic food records
Queiroz et al, 2022	Brazil	50% carbohydrate, 20% protein, 30% fat	Prescribe daily meals	Not report	Participants sent photos to the nutritionist researcher through a smartphone app before consuming the food
Ferrocino et al, 2022	Italy	45–55% carbohydrates, <10% sugars, 30% fats, 15–25% proteins, 20–30 g fiber	Verbal and written advice on practical lifestyle tips.	Moderate activity, such as brisk walks for at least 150 min/week, plus 30 min/week of exercise against resistance, was recommended	weigh foods to ensure both adherence to the given recommendations and accurate reporting of their caloric intake
Jamshed et al, 2022	Pakistan	not report	one-on-one counseling from registered dietitian for 30-minute	exercise 75 to 150 min/wk based on baseline activity	report their eating window through a software and classify as adherent if they follow their assigned eating window within 30 minutes
Zhang et al, 2022	China	No constraints	15 min instruction about completing the food records at baseline	Keep their habitual physical activity	Receive an SF-400A electronic compact scale to measure the time of foods consumed
**Crossover studies**					
Kahleova et al, 2014	Czech	55% carbohydrates, 20-25% protein, less than 30% fat, 30-40 g/day fiber	1 h weekly meetings with lectures and cooking classes	Keep their habitual physical activity	Analysis dietary records using a country-specific food-nutrient NutriDan
Sutton et al, 2018	USA	50% carbohydrate 35% fat, and 15% protein	Not report	maintain consistent physical activity	eat meals at research clinic or be supervised via remote video monitoring by Skype

The macronutrient, consultation provided, physical exercise intensity, and methodology for measuring adherence were extracted from the included articles for this meta-analysis.

Table S4. Risk of bias assessment of RCT and crossover studies. Related to Table 3.

(A). Risk of bias of RCT studies.

Study	D1	D2	D3	D4	D5	Overall
Gabel et al, 2018	Low	Low	Low	Low	Low	Low
Chow et al, 2020	Low	High	Low	Low	Low	High
Domaszewski et al, 2020	Low	High	High	Low	Low	High
Cienfuegos et al, 2020	Low	Low	Low	Low	Low	Low
Kunduraci et al, 2020	Low	Some concerns	Low	Low	Low	Some concerns
Lowe et al, 2020	Low	Low	High	Low	Low	High
Peeke et al, 2021	Low	Low	Low	Low	Low	Low
Kotarsky et al, 2021	Low	High	Low	Low	Low	High
Moro et al, 2021	Low	High	High	Low	Low	High
Ribeiro et al, 2021	Low	Some concerns	Low	Low	Low	Some concerns
Che et al, 2021	Low	Low	Low	Low	Low	Low
Lin et al, 2022	Low	Low	Low	Low	Low	Low
Liu et al, 2022	Low	Low	Low	Low	Low	Low
Thomas et al, 2022	Low	Low	Low	Low	Low	Low
Queiroz et al, 2022	Low	Low	High	Low	Low	Low
Ferrocino et al, 2022	High	Low	Low	Low	Low	High
Jamshed et al, 2022	Low	Low	Low	Low	Low	Low
Zhang et al, 2022	Low	Some concerns	Low	Low	Low	Some concerns

(B). Risk of bias of crossover studies

Study	D1	DS	D2	D3	D4	D5	Overall
Kahleova et al, 2014	Low	Low	Low	Low	Low	Low	Low
Sutton et al, 2018	Low	Low	Low	Low	Low	Low	Low

Risk of bias of included studies. The risk of bias was assessed by the Cochrane Collaboration Risk of Bias 2.0 Tool for RCTs and Risk of Bias 2.0 for crossover trials. The five assessment dimensions were explained below: (1) Randomization process: whether the allocation sequence was random and concealed; (2) Deviations from intended interventions: whether the participants and experimenters were aware of the assigned intervention (Given the nature of the intervention that could not be double-blind, we did not consider the performance bias) and whether an appropriate analysis (intention-to-treat or modified intention-to-treat) was used to estimate the intervention effect; (3) Missing outcomes: whether the outcome data available for all, or nearly all participants. Here we suggested the missing data more than 10% due to the intervention bias as high risk of bias; (4) Measurement of the outcomes: whether the outcomes measurement appropriate; (5) Selection of the reported results: whether the reported data were in accordance with a pre-specified analysis plan and whether results were selected on the bias of results from multiple measurements or analyses.

Table S5. The results of risk of bias for included studies. Related to Table 3.

(A). Results of risk of bias of RCT studies.

	D1	D2	D3	D4	D5	Overall
Gabel et al, 2018	+	+	+	+	+	+
Chow et al, 2020	+	?	+	+	+	?
Domaszewski et al, 2020	+	?	?	+	+	?
Cienfuegos et al, 2020	+	+	+	+	+	+
Kunduraci et al, 2020	+	?	+	+	+	!
Lowe et al, 2020	+	+	?	+	+	?
Peeke et al, 2021	+	+	+	+	+	+
Kotarsky et al, 2021	+	?	+	+	+	?
Ribeiro et al, 2021	+	?	+	+	+	!
Che et al, 2021	+	+	+	+	+	+
Lin et al, 2022	+	+	+	+	+	+
Liu et al, 2022	+	+	+	+	+	+
Thomas et al, 2022	+	+	+	+	+	+
Queiroz et al, 2022	+	+	?	+	+	?
Ferrocino et al, 2022	?	+	+	+	+	?
Jamshed et al, 2022	+	+	+	+	+	+
Zhang et al, 2022	+	?	+	+	+	!

(B). Results of risk of bias of crossover studies.

	D1	DS	D2	D3	D4	D5	Overall
Kahleova et al, 2014	+	+	+	+	+	+	+
Sutton et al, 2018	+	+	?	+	+	+	?

The overall bias integrated these five aspects and were coded as low (the green circle), of some concern (the yellow circle), or high (the red circle).

Table S6. GRADE assessment of all outcomes. Related to Table 3.

Outcomes	Risk of bias	Inconsistency	Indirectness[c]	Imprecision	Publication Bias	Study numbers	Overall quality of evidence[f]
WL%	Serious limitations[a]	No serious limitations	Serious limitations	No serious limitations	No serious limitations	22	⊕⊕〇〇
WC	No serious limitations	No serious limitations	Serious limitations	No serious limitations	Serious limitations[e]	11	⊕⊕〇〇
BMI	No serious limitations	No serious limitations	Serious limitations	No serious limitations	No serious limitations	14	⊕⊕⊕〇
FM	Serious limitations[a]	Serious limitations[b]	Serious limitations	No serious limitations	No serious limitations	17	⊕〇〇〇
BLM	Serious limitations[a]	Serious limitations[b]	Serious limitations	No serious limitations	No serious limitations	18	〇〇〇〇
VF	Serious limitations[a]	No serious limitations	Serious limitations	Serious limitations[d]	Serious limitations[f]	6	〇〇〇〇
EI	No serious limitations	No serious limitations	Serious limitations	No serious limitations	Serious limitations[e]	16	⊕⊕〇〇
SBP%	No serious limitations	Serious limitations[b]	Serious limitations	No serious limitations	No serious limitations	13	⊕⊕〇〇
DBP%	No serious limitations	No serious limitations	Serious limitations	Serious limitations[d]	No serious limitations	13	⊕⊕〇〇
HR	No serious limitations	Serious limitations[b]	Serious limitations	Serious limitations[d]	Serious limitations[f]	8	〇〇〇〇
TG	No serious limitations	No serious limitations	Serious limitations	Serious limitations[d]	No serious limitations	17	⊕⊕〇〇
TC	No serious limitations	Serious limitations[b]	Serious limitations	Serious limitations[d]	No serious limitations	16	⊕〇〇〇
HDL	Serious limitations[a]	Serious limitations[b]	Serious limitations	Serious limitations[d]	No serious limitations	18	〇〇〇〇
LDL	No serious limitations	Serious limitations[b]	Serious limitations	Serious limitations[d]	Serious limitations[e]	16	〇〇〇〇
FG	No serious limitations	Serious limitations[b]	Serious limitations	No serious limitations	Serious limitations[e]	18	⊕〇〇〇
FI	No serious limitations	Serious limitations[b]	Serious limitations	No serious limitations	Serious limitations[e]	16	⊕〇〇〇
HOMA-IR	No serious limitations	No serious limitations	Serious limitations	No serious limitations	No serious limitations	13	⊕⊕⊕〇
HbA1c	No serious limitations	Serious limitations[b]	Serious limitations	Serious limitations[d]	Serious limitations[e]	10	〇〇〇〇
RMR	Serious limitations[a]	No serious limitations	Serious limitations	Serious limitations[d]	Serious limitations[f]	5	〇〇〇〇

a: The proportion of information from results at high risk of bias in RoB 2.0 assessment is sufficient to affect the interpretation of results.

b: High heterogeneity (I^2 >50%) that not can be explained by energy prescription or eating time of day.

c: All outcomes are substitutes for the patient-important metabolic outcomes of interest.

d: The 95% confidence interval is so wide that it overlaps no effect and the appreciable benefit threshold. The threshold was calculated as estimate effect increase of 25%.

e: Publication bias because there may be the equivalent number of 'negative' trials that have not been included in this analysis.

f: Few study trials.

g: evidence of high certainty: ⊕⊕⊕⊕, evidence of moderate certainty: ⊕⊕⊕〇, evidence of low certainty: ⊕⊕〇〇, evidence of very low certainty: ⊕〇〇〇

We evaluated the quality of evidence as high, moderate, low, and very low using the Grading of Recommendations, Assessment, Development, and Evaluation (GRADE) method in terms of risk of bias, inconsistency, indirectness, imprecision, and publication bias. Two authors (the first and second author) defined the specific criteria for judging these five aspects and inconsistencies were discussed and resolved by the third author.
